# Environmental Heat Exposure Among Pet Dogs in Rural and Urban Settings in the Southern United States

**DOI:** 10.3389/fvets.2021.742926

**Published:** 2021-10-05

**Authors:** Katherine E. Moon, Suwei Wang, Kaya Bryant, Julia M. Gohlke

**Affiliations:** ^1^Department of Population Health Sciences, Virginia Tech, Blacksburg, VA, United States; ^2^Translational Biology, Medicine and Health (TBMH), Virginia Tech, Roanoke, VA, United States; ^3^College of Veterinary Medicine, Tuskegee University, Tuskegee, AL, United States

**Keywords:** canine, heat-related illness, environment, individually experienced temperature, rural, pet dog, heat stress

## Abstract

With advancing global climate change, heat-related illnesses and injuries are anticipated to become more prevalent for humans and other species. Canine hyperthermia is already considered an important seasonal emergency. Studies have been performed on the risk factors for heat stroke in canine athletes and military working dogs; however there is limited knowledge on environmental risk factors for the average pet dog. This observational study explores variation in individually experienced environmental temperatures of pet dogs (*N* = 30) in rural and urban environments in central Alabama. Temperature data from dogs and their owners was collected using wearable personal thermometers. Demographic data on the dogs was collected using a brief survey instrument completed by their owners. Dogs included in the study varied in signalment, activity level, and home environment. Linear mixed effects regression models were used to analyze repeated measure temperature and heat index values from canine thermometers to explore the effect of environmental factors on the overall heat exposure risk of canine pets. Specifically, the heat exposures of dogs were modeled considering their owner's experienced temperatures, as well as neighborhood and local weather station measurements, to identify factors that contribute to the heat exposure of individual dogs, and therefore potentially contribute to heat stress in the average pet dog. Results show hourly averaged temperatures for dogs followed a diurnal pattern consistent with both owner and ambient temperature measurements, except for indoor dogs whose recordings remained stable throughout the day. Heat index calculations showed that owners, in general, had more hours categorized into the National Weather Station safe category compared to their dogs, and that indoor dogs had a greater proportion of hours categorized as safe compared to outdoor dogs. Our results suggest that the risk of the average pet dog to high environmental heat exposure may be greater than traditional measures indicate, emphasizing that more localized considerations of temperature are important when assessing a dog's environmental risk for heat-related injury or illness.

## Introduction

Global climate change is a One Health crisis, threatening both veterinary and medical public health (1, 2). Expanding exposure to increasingly high environmental temperatures is a major concern in the coming decades (3). Extreme heat events are already responsible for the highest counts of natural disaster-related morbidity and mortality (4, 5). Increased attention is therefore necessary to identify and implement adaptation strategies. Previous work has explored changing heat exposure levels in humans and increased risk for heat strain and stroke (6–8). These same risks apply to our companion and livestock animals. Studies have attempted to quantify the effects of exertion, environmental temperature and individual factors that augment individual risk for heat related illness or injury in military working dogs, canine athletes, and production animals (9–12). However, limited attention has been given to the environmental factors that contribute to the average pet dog's risk for heat related illness or injury.

As in humans, canine heat stroke occurs when an individual's heat dissipation mechanisms are overwhelmed (13, 14). Risk factors that lower the threshold for heat stroke can be individual or environmental and include body condition score, breed, coat type, respiratory capacity, fitness level, hydration status, ambient temperature, and relative humidity (13). Heat stroke can occur quickly and have serious and lasting consequences (15). Traditional guidance given to owners to prevent heat stroke in their pets is vague and generally based on human experiences of heat, unvalidated for their canine companions (11, 16). Despite this, there is evidence suggesting that dogs are less tolerant of high temperature and high humidity compared to humans (17). Specific guidelines and models of heat exposure for dogs that do exist are generally based on studies involving a few specific breeds, working in highly specific environments (i.e., military working dogs (MWD), Federal Aviation Administration transportation studies, canine athletes) (11, 18, 19). Although several case studies have examined the environmental factors prevalent in dogs presenting or diagnosed for heat stroke (or death) retrospectively, we are unaware of any studies that have reported the daily heat exposure of the average pet dog using individually experienced temperatures.

Heat exposure can be estimated using various means including monitoring of physiological, behavioral, and environmental factors. Although physiological parameters may provide a more detailed picture of an individual animal's response to their environment, they often require invasive, expensive, or impractical measures for monitoring in the field (or home) (20), reducing feasibility for use in field-based epidemiological studies. Ambient temperature can be monitored using small thermometers (such as iButtons^®^) placed on an individual animal's collar. Studies in both humans and animals have demonstrated their reliability as an estimate of individually experienced temperature and body temperature (6, 20–22). They are minimally invasive, inexpensive, and easily distributed to study participants. Additionally, in the context of heat exposure, they allow for more precise measurement than traditional means, where data collected from an oftentimes far away weather station is used to calculate the National Weather Service's heat index (23). Evidence shows that there is wide variability in temperature and climate within neighborhoods, which would strongly impact an individual's experience of heat (24–26).

Here we present a descriptive study demonstrating feasibility and utility of measuring individually experienced temperatures of pet dogs in both an urban and rural setting. Data for this study was obtained in conjunction with another project designed to measure human personal heat exposures in urban and rural environments (27). A subset of participants in that project volunteered to also collect data from their pet dogs. The objectives of the study were (1) to determine the feasibility of a small thermometer clipped to a dog's collar as a measure of heat exposure in pet dogs and (2) explore home and neighborhood level risk factors for increased heat exposure in pet dogs.

## Methods

### Study Recruitment and Temperature Data Collection

Data for this analysis were retrieved from a study performed in conjunction with an investigation into heat exposure of rural and urban outdoor workers and residents (22, 27, 28). Participants with a dog were invited to enroll their dog in a parallel study in which both owner and pet wore thermometers (on the owner's shoe and the dog's collar) for 7 days. Owners came to a training session in which they practiced taking the iButton on and off shoes and suggested places to clip on different types of shoes were reviewed. Owners were instructed to place their shoes with the iButton clipped to them or the unclipped iButton by their bed at nighttime. If a participant had more than one dog, the participant was given the opportunity to enroll only one of the dogs into the study. iButton^®^ thermometers (model #DS1922L) (Maxim Integrated, California, USA) contain a computer chip within a 16 mm thick steel casing, have a temperature resolution of ± 0.5°C for temperatures from −10 to 65°C, and have an operating range of −40 to 85°C (29). Owners were instructed on proper iButton orientation (face down to avoid direct sunlight) and collar fit on their dogs, and a follow up call was made halfway through the study period to confirm compliance and answer any questions concerning the study. Owners were asked not to remove collars at nighttime or any other time during the study period. Entry into the study was staggered, and iButtons were set to record temperature readings every 5 min for 7 days during the study period of July 10–19, 2017. Owners received $150 to participate in the main study and were compensated an additional $3 a day (maximum of $15) and a new dog collar was provided in exchange for participating in the dog study, which included completing the owner survey, attaching the iButton to the dog, and returning the iButton at the end of the study. The owner survey collected information on the dog's signalment, body weight and body condition, medical conditions, coat characteristics (color, length, fur type), activity level, exercise frequency and duration, home environment, and estimated number of hours spent outside per day. Individually experienced temperature data collected from the owners, their local neighborhoods, and local weather stations was obtained following methods described previously (22). Briefly, human participants wore an iButton attached to their shoe for the duration of the 6-day study, shielded iButtons with a thermometer and hygrometer were placed outdoors within neighborhoods of participants to capture neighborhood level temperature and humidity, and data collected by weather stations used for local forecasting were also included in the analysis as a comparison. Weather stations are typically housed at the nearest airport and may not capture locally experienced temperatures. Virginia Tech Institutional Review Board (VT-IRB protocol # 17-068) and Virginia Tech Institutional Animal Care and Use Committee (VT-IACUC protocol # 17-134) approved the protocols for human and pet participants, respectively.

### Survey and Temperature Data Processing

Survey data collected from the dog owners were assessed for readability and completeness and variables with insufficient numbers of responses were not analyzed further. This study focused on variables related to environmental heat exposures. A new variable describing the home environment of the dog was established based on responses of “Dog Living Place” and “Dog Outside Duration.” Dogs marked as “Indoor/Outdoor” for “Dog Living Place” were recategorized as “Indoor” if “Dog Outside Duration” was ≤12 h, and “Outdoor” if “Dog Outside Duration” was >12 h. Survey variables used in the final analysis included “Rural/Urban” and “Indoor/Outdoor.” “Rural/Urban” was determined by the study location [City of Birmingham (urban) or Wilcox County (rural)] in which the dog's owners resided.

Temperature data from the dog iButtons was downloaded to password-protected computers, assigned a numeric Dog ID, and linked to their owner's iButton temperature and survey data, as well as their respective neighborhood temperature data (as determined by the home address given by the dog owner). Temperature measurements recorded outside the individual dog's 6-day study period were excluded. To account for the extra time owner's may have taken to fasten the iButton to their dog's collar and for early removal prior to turn-in, a dog's study period was defined as starting at 12 am the morning after the owner received the iButton and concluding at 12 am the night before turn-in.

Upper outliers were removed from temperature data to eliminate potential iButton artifacts. The Modified Absolute Median method (30) with a cutoff of 3.5°C was used for each location and home environment. After outlier removal, 5 min temperature data were rounded to the nearest hour using the “round_date” function in “lubridate” package in R (31). These values were than averaged for each hour of the study period.

### Correction Factor Applied to Canine Pet Temperature Data

Canine temperature data was adjusted to reduce the contribution of body heat to the recorded temperatures. The correction factor was developed using data from an indoor, medium coat length, mixed breed pet dog living in a supervised environment. The measurements were compared hourly to data collected from iButtons recording temperatures in two rooms normally inhabited by the dog. The hourly average temperatures of the two rooms were averaged to approximate an indoor ambient air temperature. This ambient air temperature was used to calculate the average hourly difference in temperatures between the ambient air and the dog iButton, thus approximating the hourly contribution of body heat. These hourly differences were averaged over 4 six-hour periods corresponding to varying dog activity levels in the early morning. The calculated correction factors were: −3.9°C for early morning (4:00–9:59 AM), −3.0°C for daytime (10:00 AM−3:59 PM), −2.0°C for evening (4:00–9:59 PM), and −4.4°C for night (10:00 PM−3:59 AM). As a sensitivity analysis, we also provide results using only two correction factor periods (7:00 AM to 9:59 PM and 10:00 PM to 6:59 AM) in Supplementary Material 1. We would expect the pattern of temperature differences would vary across dogs based on individual dog and owner routines.

### Combining Canine Pet Data With Owner, Neighborhood, WS Data

Temperature taken by thermometers and access to air conditioning data from dog owners, and temperature and relative humidity data from the nearest neighborhood monitors and the nearest local weather stations underwent similar data processing procedures and is described in detail elsewhere (22). Data from canine pets was simultaneously matched by date and time of day with their corresponding owner, nearest neighborhood monitor, and nearest weather station.

### Data Analysis

Descriptive statistics were performed on the stratified datasets (by temperature source, location, and home environment) and diurnal patterns were graphed using “ggplot2” package in R using hourly mean temperatures (32).

Heat Index calculations were performed by matching the canine hourly averaged temperatures with the nearest weather station's relative humidity and applying the ‘weathermetrics' package in R (33). Using the heat index values generated, Risk Levels were assigned to each dog's hourly heat exposure using the National Weather Service categories: Safe [<27°C (80°F)], Caution [27–32°C (80–90°F)], Extreme Caution [32–39°C (90–103°F)], Danger [40–51°C (103–124°F)], and Extreme Danger [≥52°C (125°F)] (37).

Linear mixed effects regression models (“lmer” function from “lme4” package in R) were used to assess the associations of location and home environment to heat exposure of pet dogs (34). Additionally, owner, neighborhood, and weather station temperature data were added sequentially to the model to assess their contribution to heat exposure of pet dogs. Confidence intervals were calculated, and Akaike Information Criterion (AIC) values were assessed for comparison of the linear mixed models.

## Results

Fifteen dogs were recruited from each study site for a total of thirty dogs. Selected demographics and survey answers are summarized in Table 1. The mean estimated age of the dogs was 5.7 years with a range of 7 months to 25 years reported by the owners. Fourteen (14/30; 46.7%) of the dogs were female and were close to evenly split between rural and urban locations. Rural dogs were estimated by their owners to spend more time outside with an average of 17.9 h compared to an average of 11.0 h for urban dogs. Similarly, more urban dogs were reported to be indoor dogs (8/15; 53.3%) than rural dogs (3/15; 20.0%). After using reported hours outside and home environment to dichotomize a dog's indoor/outdoor status, 14 (46%) study dogs were labeled indoor, with 10 (66.7%) located in the urban environment and 4 (26.7%) located in the rural environment. No dogs were reported by their owners as having health conditions that would significantly impact their ability to tolerate hot and humid conditions.

**Table 1:** Canine demographics and characteristics.

**Group**	**Combined**	**Urban setting**	***p*-value**	**Rural setting**
Participant number	30 (100%)	15 (50%)		15 (50%)
Mean age (range), years	5.7 (0.6–25)	6.1 (1–25)	0.76	5.3 (0.6–17)
Missing data	4 (13.3%)	2 (13.3%)		2 (13.3%)
Sex-Female	14 (46.7%)	8 (53.3%)	0.57	6 (40.0%)
Missing data	1 (3.3%)	0 (0%)		1 (6.7%)
Neutered- Yes	10 (33.3%)	3 (20.0%)		7 (46.7%)
Missing data	3 (10.0%)	3 (20.0%)		0 (0%)
Weight (mean, range), kg	17.0 (1.8–45.4)	16.2 (1.8–45.4)	0.44	12.5 (2.3–45.4)
Missing data	3 (10.0%)	1 (6.7%)		2 (13.3%)
Hours outside (mean, range)	14.5 (1–24)	11.0 (1–24)	0.09	17.9 (1–24)
Missing data	2 (6.7%)	1 (6.7%)		1 (6.7%)
Home environment			0.15	
Indoor only	11 (36.7%)	8 (53.3%)		3 (20.0%)
Outdoor only	14 (46.7%)	5 (33.3%)		9 (60.0%)
Indoor/outdoor	3 (10.0%)	1 (6.7%)		2 (13.3%)
Missing data	2 (6.7%)	1 (6.7%)		1 (6.7%)
Indoor var. ^a^	14 (46%)	10 (66.7%)	2.2e-16^*^	4 (26.7%)

* *A statistically significant difference with p < 0.05*.

a *Determined using owner report of Home Environment and Hours Outside. One owner was contacted to complete dataset, as neither value was provided on the survey. One owner provided only hours outside, which was used to determine the dog's status. Indoor/Outdoor dogs were dichotomized using indoor = Hours outside ≤12, outdoor = Hours Outside >12*.

The average distance to a nearest neighborhood iButton was 5.3 km for dog owning participants in the rural location and 6.4 km for dog owning participants in the urban location (Table 2). The average distance to the nearest weather station was 46.0 km for rural dogs and 13.5 km for urban dogs. Fifteen of the 30 dog owners provided information about at home air conditioning. Of those, 8 had access to air conditioning, 3 from the rural location compared to 5 from the urban location.

**Table 2:** Distance between owner home and the nearest neighborhood monitor and the nearest WS.

**Group**	**Temperature source**	**Mean (95% confidence interval) distance to participant home, km**
Rural owners	Neighborhood monitor	5.3 (5.1–5.5)
	WS	46.0 (45.8–46.3)
Urban owners	Neighborhood monitor	6.4 (6.1–6.7)
	WS	13.5 (13.3–13.7)

### Temperature Data Descriptive Statistics

**Figure 1:**
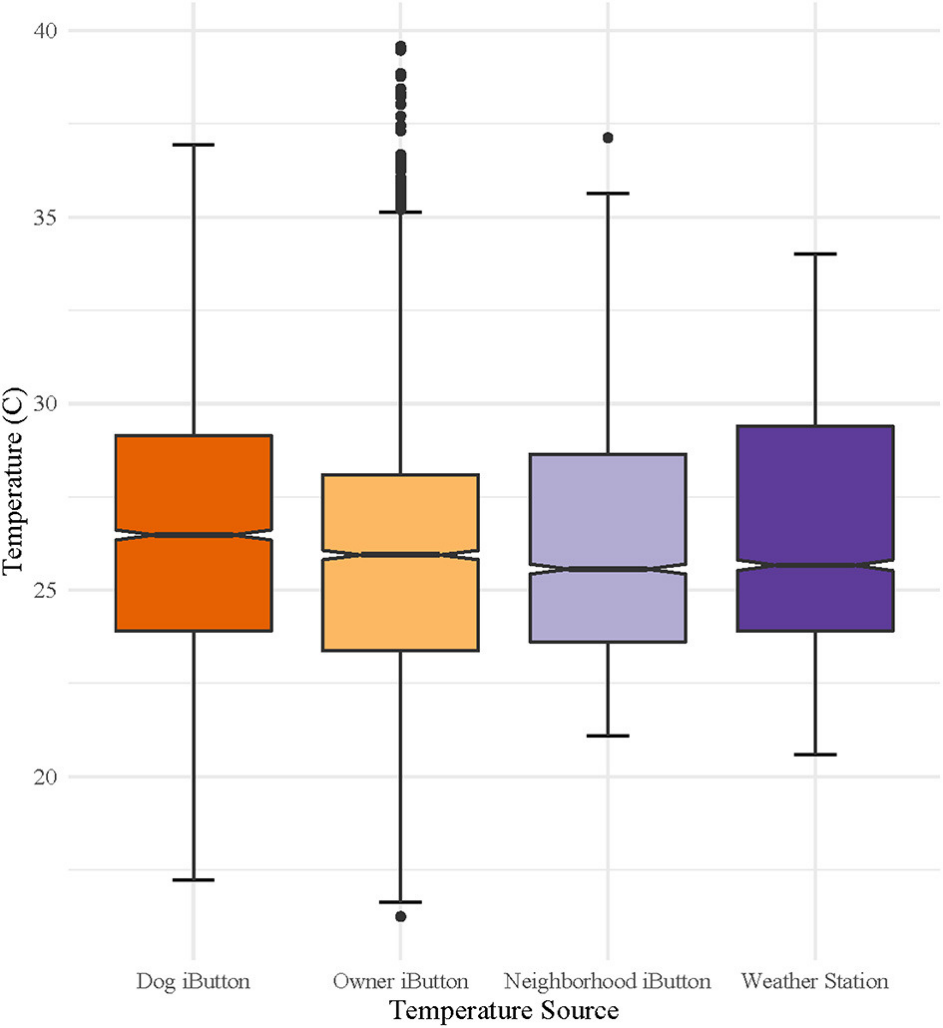
Median and range of temperatures measured on canine pets, their owners, neighborhood thermometers, and the nearest weather station. Canine pet and owner data were collected over 6-day period for each dog, with staggered enrollment over a 10-day period in July 2017, using wearable iButton^®^ thermometers. Neighborhood ambient temperatures were captured using shielded iButton^®^ thermometers/hygrometers placed in neighborhoods and weather station data was downloaded from the National Weather Service weather station data archive.

**Figure 2:**
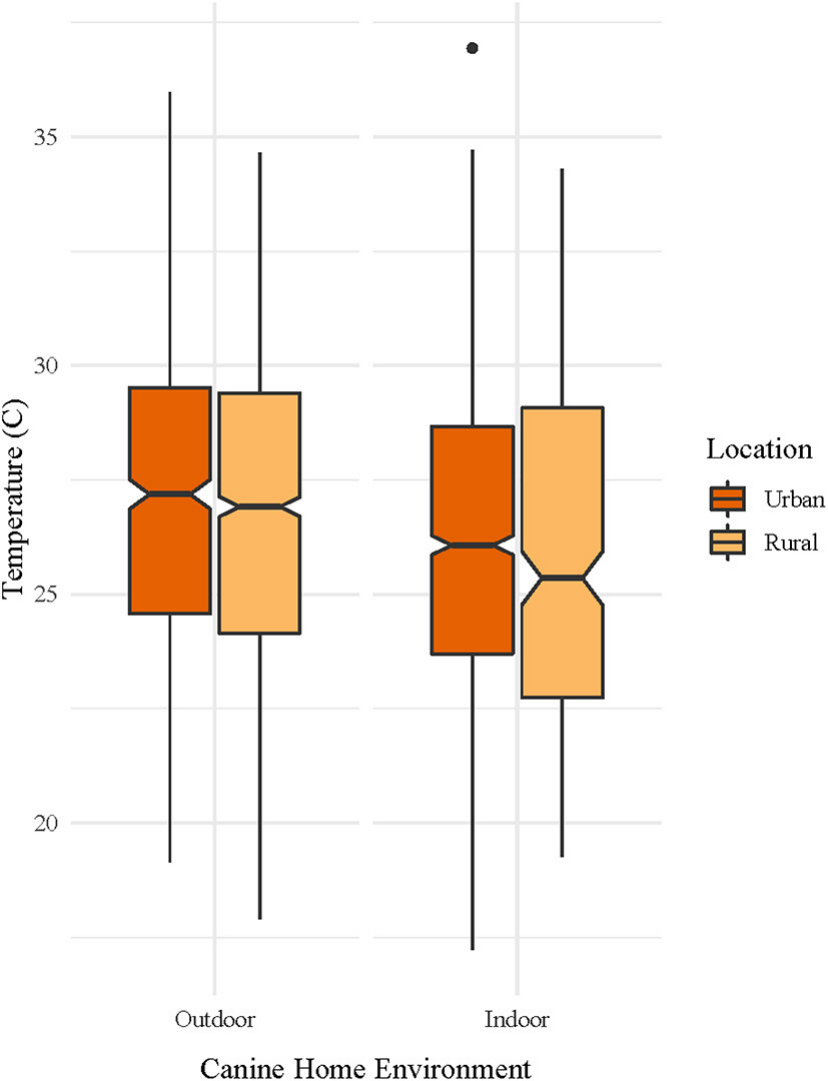
Median and range of temperatures measured on canine pets, stratified by home environment (outdoor or indoor) and location (urban or rural). Canine pet and owner data were collected over a 6-day period for each dog, with staggered enrollment over a 10-day period in July 2017, using wearable iButton^®^ thermometers. Neighborhood ambient temperatures were captured using shielded iButton^®^ thermometers/hygrometers placed in neighborhoods and weather station data was downloaded from the National Weather Service weather station data archive.

Of the thirty iButton thermometers distributed to dog owners, 26 were returned with usable data. A total of 882 observations out of 51,480 observations were removed as outliers. Figure 1 shows the distribution of temperatures measured by iButtons for each source (Dog, Owner, Neighborhood, and Weather Station). Overall, median adjusted temperatures recorded by dog thermometers (26.6°C) were higher than those recorded by owner thermometers (25.9°C), neighborhood monitors (25.6°C) and weather station monitors (25.6°C), respectively. Figure 2 shows median and ranges of temperatures recorded on dogs, stratified by location (urban or rural) and home environment (indoor or outdoor). Temperatures from rural and urban dogs were relatively similar in distribution pattern, although urban dogs experienced higher maximum temperatures, 36.9°C vs. 34.7°C, but lower median temperatures, 26.3°C vs. 26.7°C. Indoor dogs experienced an overall cooler median temperature than outdoor dogs, 25.8°C vs. 27.4°C, although the maximum temperature experienced by both groups were similar at 35.6°C and 35.3°C, respectively.

### Diurnal Patterns of Heat Exposure

**Figure 3:**
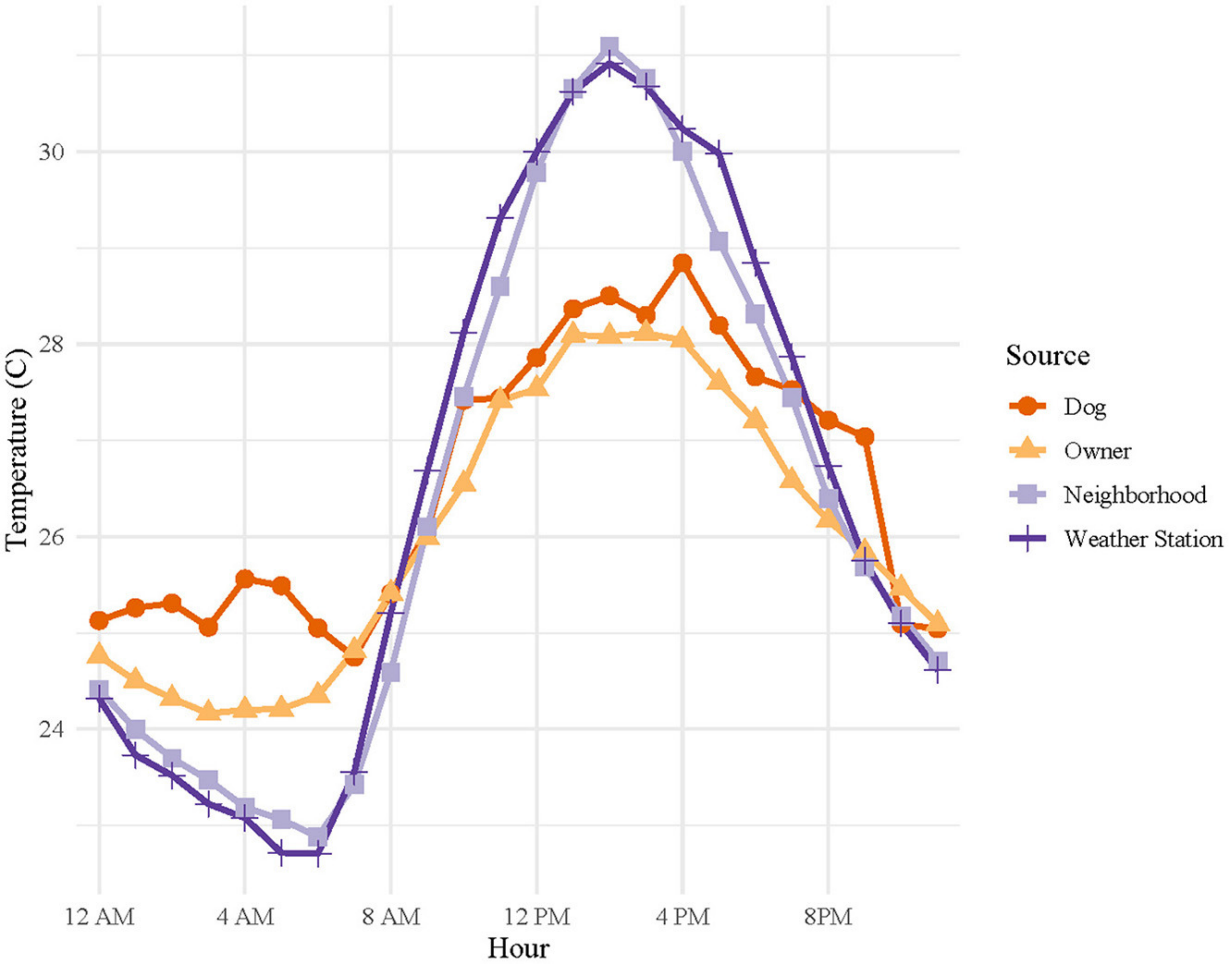
Diurnal variation of mean hourly temperatures for all canine pets' thermometers, owners' thermometers, neighborhood thermometers, and nearest weather station. Canine pet and owner data were collected over a 6-day period for each dog, with staggered enrollment over a 10-day period in July 2017, using wearable iButton^®^ thermometers. Neighborhood ambient temperatures were captured using shielded iButton^®^ thermometers/hygrometers placed in neighborhoods and weather station data was downloaded from the National Weather Service weather station data archive.

Using the average hourly temperature for each source (dog, owner, neighborhood, or weather station), we plotted the diurnal variation in temperatures measured (Figure 3). For all temperature sources, a clear diurnal pattern was discernable. Between 8 AM and 8 PM, weather station and neighborhood measurements overestimate the average temperatures experienced by owners and their dogs. From 8 PM to 8 AM, environmental measurements underestimate average individually experienced temperatures of owners and dogs.

**Figure 4:**
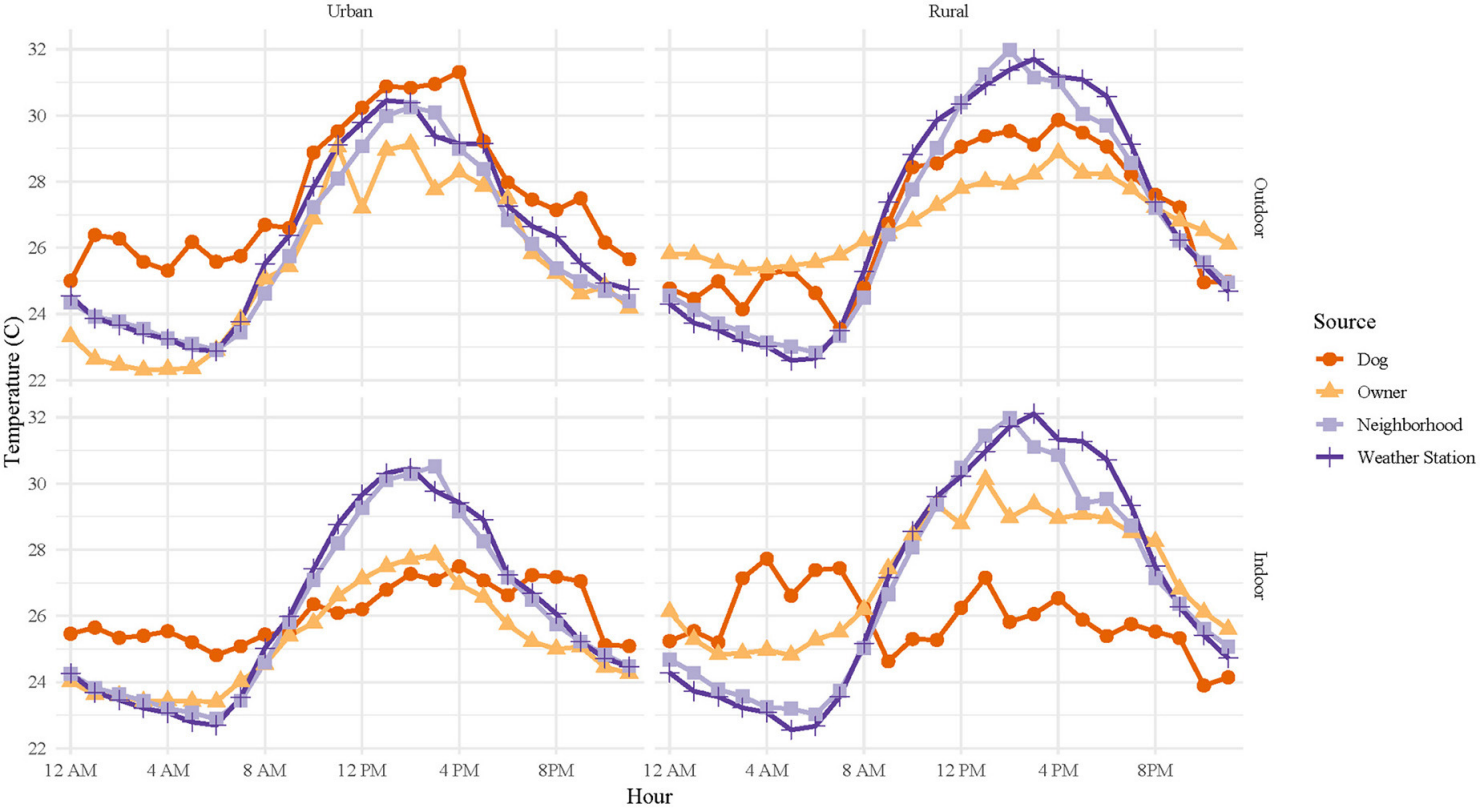
Diurnal variation of mean hourly temperatures for all canine pets' thermometers, owners' thermometers, neighborhood thermometers, and nearest weather station, stratified by location (urban or rural) and home environment (outdoor or indoor). Canine pet and owner data were collected over a 6-day period for each dog, with staggered enrollment over a 10-day period in July 2017, using wearable iButton^®^ thermometers. Neighborhood ambient temperatures were captured using shielded iButton^®^ thermometers/hygrometers placed in neighborhoods and weather station data was downloaded from the National Weather Service weather station data archive.

When stratified by dog home environment, the diurnal pattern is lost for indoor dogs, with average daily temperature variation ranging ~0–2°C compared to ~0–6°C for outdoor dogs (Figure 4). When stratified by location, diurnal patterns are similar for both rural and urban dogs (Figure 4). However, we do see that dog owners' thermometers recorded temperatures ~1.5–2°C cooler than their adjusted dogs' thermometers in both urban and rural locations.

### Heat Risk Level Classification

**Figure 5:**
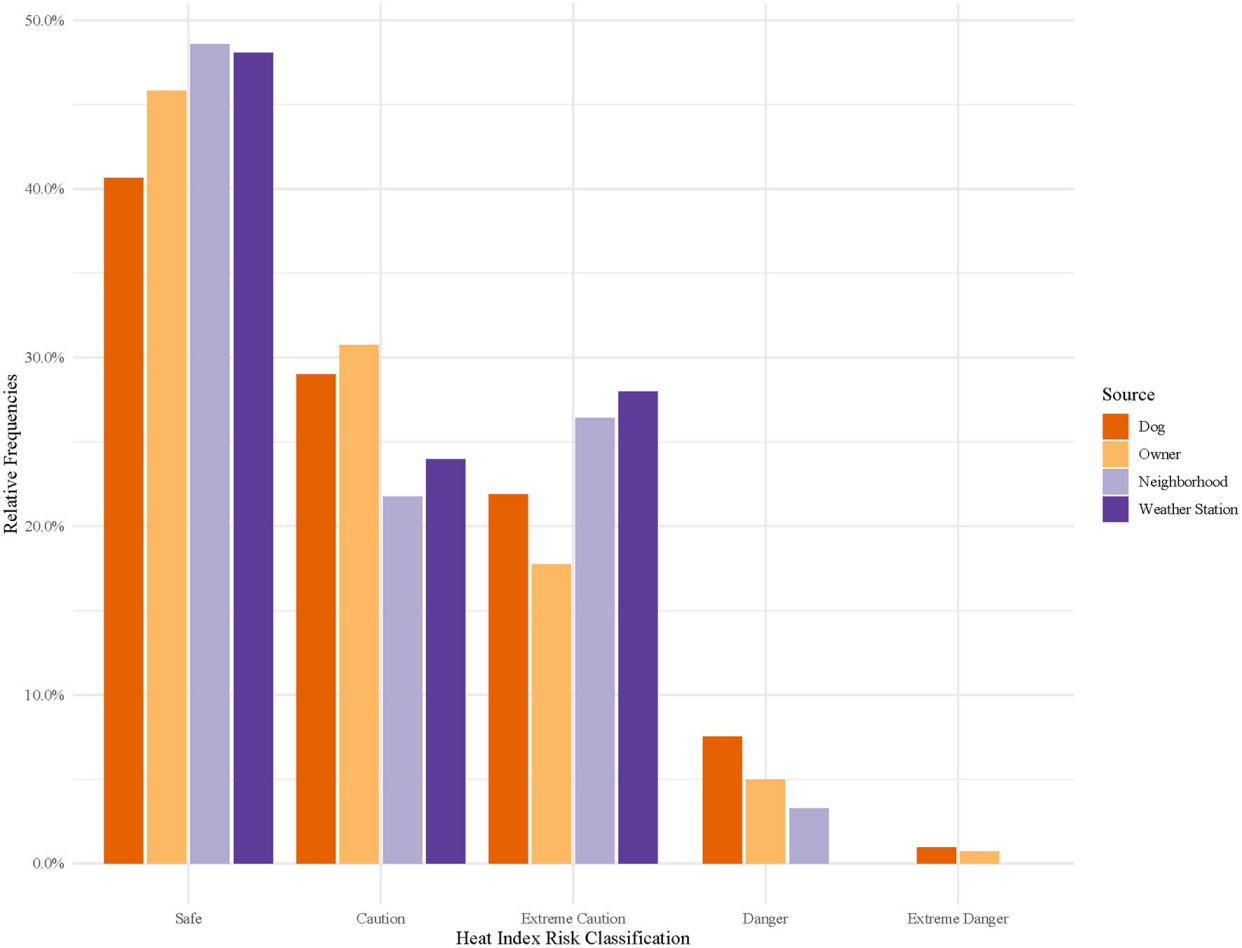
Relative frequencies of hourly heat index risk classification calculated for canine pets' thermometers, owners' thermometers, neighborhood weather stations, and nearest weather station. Heat index risk classifications were determined by the National Weather Service's designations: Safe (<80°F), Caution (80–90°F), Extreme Caution (90–103°F), Danger (103–124°F), and Extreme Danger (≥125°F) (35).

**Figure 6:**
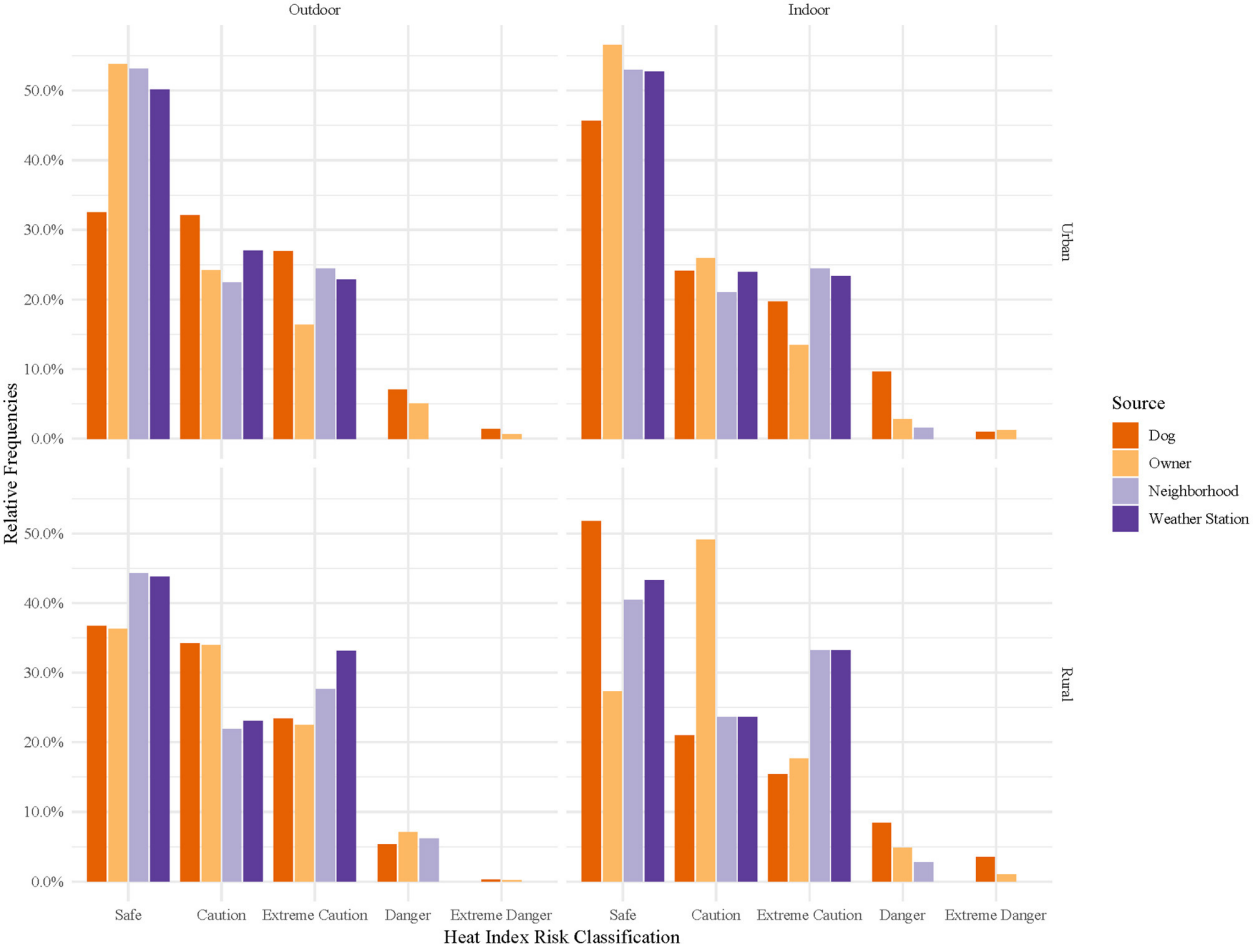
Relative frequencies of hourly heat index risk classification calculated for canine pets' thermometers, owners' thermometers, neighborhood weather stations, and nearest weather station, stratified by home environment (outdoor or indoor) and location (urban or rural). Heat index risk classifications were determined by the National Weather Service's designations: Safe (<80°F), Caution (80–90°F), Extreme Caution (90–103°F), Danger (103–124°F), and Extreme Danger (≥125°F) (35).

**Figure 7:**
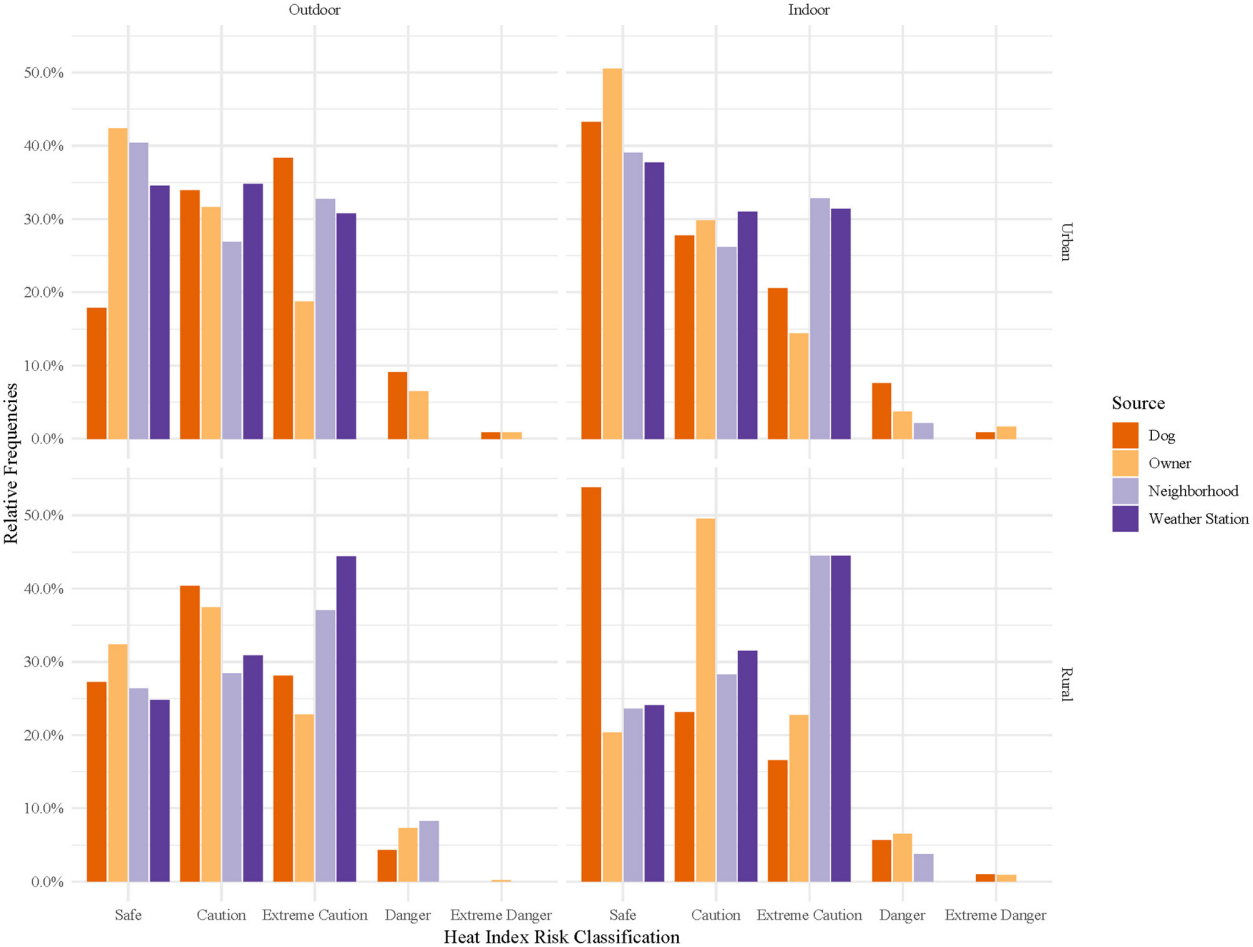
Relative frequencies of hourly heat index risk classification during daytime hours as calculated for canine pets' thermometers, owners' thermometers, neighborhood weather stations, and nearest weather station, stratified by home environment (outdoor or indoor) and location (urban or rural). Daytime hours were 6 AM to 11 PM. Heat index risk classifications were determined by the National Weather Service's designations: Safe (<80°F), Caution (80–90°F), Extreme Caution (90–103°F), Danger (103–124°F), and Extreme Danger (≥125°F) (35).

**Figure 8:**
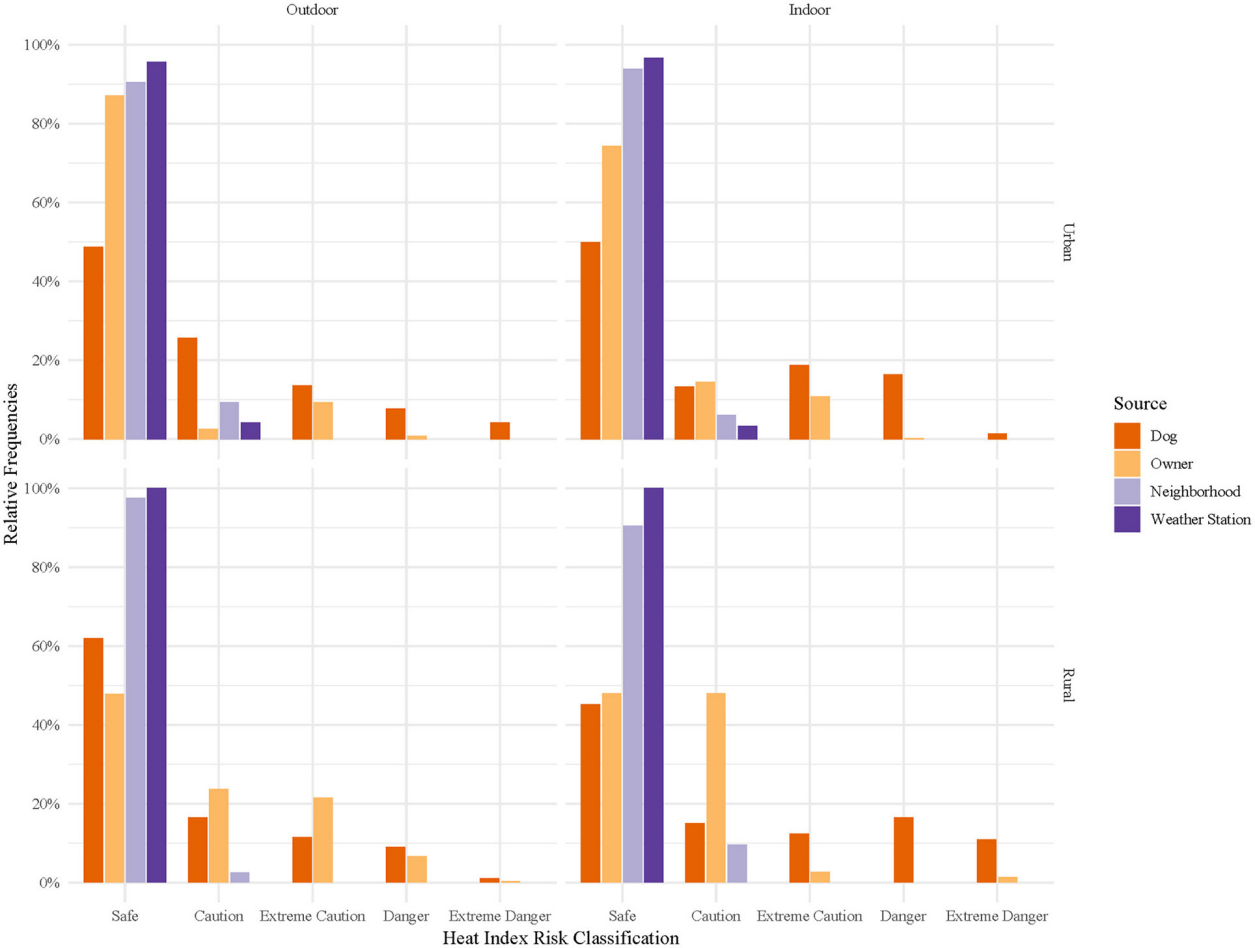
Relative frequencies of hourly heat index risk classification during nightime hours as calculated for canine pets' thermometers, owners' thermometers, neighborhood weather stations, and nearest weather station, stratified by home environment (outdoor or indoor) and location (urban or rural). Nightime hours were 12 AM to 5 AM. Heat index risk classifications were determined by the National Weather Service's designations: Safe (<80°F), Caution (80–90°F), Extreme Caution (90–103°F), Danger (103–124°F), and Extreme Danger (≥125°F) (35).

Hourly heat index classifications were compared across temperature sources (Figure 5), stratified by location and home environment (Figure 6), and time of day (Figures 7, 8). In general, owner, neighborhood, and weather station heat index classifications corresponded poorly with canine heat index risk classifications. Dogs had approximately 5–10% fewer hours in the “Safe” classification compared to all other sources. Weather station calculated heat index classifications overestimate the relative frequency of “Safe” hours recorded by dogs and do not account for any hours in either the “Danger” or “Extreme Danger” categories. In contrast, 8% of hours recorded by dogs were classified in the “Danger” or “Extreme Danger” categories. The relative frequency of “Safe” hours was 10% greater in hours measured by indoor dogs vs. outdoor dogs (Figure 6). At night, the relative frequencies of heat index classifications calculated from weather station and neighborhood iButtons differed starkly from those of dogs and their owners (Figure 8). Close to 30% of hours recorded by dogs (~15% of owner hours) corresponded to “Extreme Caution” or a more severe classification compared to 0% for both environmental temperature sources.

### Linear Mixed Effects Models of Heat Exposure

We explored the predicative ability of temperature measurement source (owner, weather station, or neighborhood monitor), location, and home environment for dog heat exposure. The results of a linear mixed model are shown in Table 3. Daily mean temperatures of owners' thermometers were negligibly lower compared to their dogs' thermometer temperatures [−0.02°C, 95% Confidence Interval (CI) −0.06 to 0.02]. On average, both weather stations and neighborhood iButtons recorded warmer daily mean temperatures than dogs' thermometers at 0.3°C (95% CI 0.2 to 0.4) and 0.1°C (95% CI, 0.07 to 0.2). On average, the model predicts that living in a rural or indoor environment provides cooler daily mean temperatures from dog thermometers, calculated at −0.8°C (95% CI −2.2, 0.7) and −1.2°C (95% CI −2.6, 0.2), respectively. A similar pattern was seen with the heat index model, with the exception that the weather station underestimated heat index by −0.05°C (95% CI −0.06 to 0.2) (Table 4).

**Table 3:** Linear mixed effect model results with dog's individually experienced temperatures over 24 h as the outcome.

**Population**	**All**
Fixed effects	β (95%CI)
Intercept	16.5 (14.9, 18.0)
T[owner] (°C)	−0.02 (−0.06, 0.02)
T[WS] (°C)	0.30 (0.23, 0.36)^*^
T[neighborhood] (°C)	0.14 (0.07, 0.21)^*^
Rural setting	−0.75 (−2.2, 0.7)
Indoor	−1.2 (−2.6, 0.22)
Rural setting: Indoor	0.25 (−2.1, 2.6)

* *A β estimate with a 95% confidence interval that does not contain 0. T, Temperature; WS, Weather Station*.

**Table 4:** Linear mixed effect model results with dog's individually experienced HI in 24 h as the outcome.

**Population**	**All**
Fixed effects	β (95%CI)
Intercept	28.3 (25.3, 31.3)
HI[owner] (°C)	−0.05 (−0.09, −0.03)^*^
HI[WS] (°C)	−0.05 (−0.06, 0.15)
HI[neighborhood] (°C)	0.11 (0.02, 0.19)^*^
Rural setting	−1.6 (−4.6, 1.4)
Indoor	−1.7 (−4.8, 1.3)
Rural setting: Indoor	1.3 (−3.7, 6.3)

* *A β estimate with a 95% confidence interval that did not contain 0. HI, Heat Index; WS, Weather Station*.

## Discussion

The research presented herein demonstrates the feasibility of collecting temperature exposure measures from dogs using an iButton clipped to the collar and describes the patterns of heat exposure in a small population of pet dogs. We also characterize environmental factors influence on heat exposure of pet dogs, including the dog's home environment and geographical location, and how similar dog's experienced temperatures are compared to temperatures experienced by their owners, and temperatures recorded within their neighborhood and at the nearest weather station. Our study demonstrates the potential use of iButton thermometers to document heat exposure patterns in pet dogs. Although iButtons have previously been used in veterinary species as a non-invasive means of approximating body temperature (20), we were able to demonstrate that with appropriate adjustments measured temperatures can also be used as a proxy for individually experienced ambient temperatures, as has been done for humans. From these data, a basis for understanding patterns of heat exposure in pet dogs (and their owners) can be obtained. This information can help inform veterinarian recommendations to clients to prevent heat-related illnesses in pet dogs.

In our study population, distinct patterns in heat exposure were discernable. Urban, outdoor dogs experienced the highest heat exposures over a 24-h period, despite rural weather station temperatures recording higher median temperatures, 26.2°C vs. 25.6°C, and higher maximum temperatures, 34.5°C vs. 32.8°C, than urban weather stations. As expected, indoor dogs had more consistent temperature patterns, lacking a clear diurnal pattern, as was seen in outdoor dogs and in dog owners. These findings suggest that local environmental conditions have a large role in the individually experienced temperatures of dogs. The lack of significance in the models for these characteristics may be due to the small size of our study populations and the uneven distribution of indoor and outdoor dogs between the rural and urban environments. However, we do see that both neighborhood and weather station measurements were more predictive of dog temperature measurements than were their owner's temperature measurements. Given that owners were unlikely to be at home with their dogs during the day, this provides support for the significance of using individually experienced temperatures to document heat exposure in pet dogs.

Obesity and reduced activity have been identified as key risk factors for heat-related illness in pet dogs (36), and the interaction between body condition and skull shape has been identified as a factor influencing heat stress and thermoregulation in brachycephalic dogs, in particular, at temperatures as low as 22°C (37). For the average pet dog, we have no systematic means of categorizing their risk from heat exposure as we do for ourselves and certain species of working animals (e.g., military working dog and dairy cattle) (8, 10, 12, 19). Using the risk measurement tool most familiar to the average pet owner, the heat index, we were able to document the relative frequency of hours in each National Weather Service Heat Index risk classification for pet dogs. As seen in Figure 5, both owners and their dogs experienced a greater number of hours in Danger and Extreme Danger classifications than as predicted by weather station heat index calculations. This was especially true at night when many may assume the risks from heat exposure are reduced. Indoor dogs, again, generally, experienced reduced heat exposure compared to outdoor dogs. However, the exception was at night, when they experienced roughly twice as many hours in “Danger” and “Extreme Danger” classifications. Explanations for this result may be reduced indoor airflow at night results in greater heat retention in homes, indoor crating at nighttime, or a higher likelihood of body position to impact the temperature recorded in indoor vs. outdoor dogs. Although our study looks at a small subset of the dog population, in a relatively small geographical area, it is useful to know that the risk of excessive heat exposure may be underestimated by our traditional measurements of heat risk.

We looked at two specific environmental factors available to us in the data, that might potentially impact the individually experienced temperatures of our pet dog population: location and home environment. Although stratifying our sample significantly reduced our sample sizes, logical patterns were visible, nevertheless. Rural dogs experienced greater median temperatures compared to their urban counterparts (26.7°C vs. 26.3°C), despite the study's maximum temperatures coming from urban dogs (36.9°C vs. 34.5°C). However, from the analysis of human participant data, rural residents were less likely to have central air conditioning in their homes (22). Thus, both indoor and outdoor rural dogs had the potential to be exposed to higher average temperatures. We can make two observations from this information: (1) urban outdoor dogs may be at greater risk for exposure to extreme heat; (2) rural indoor dog populations may be more likely to lack opportunities for cooling. Although most veterinarians are aware of the general needs of their client base, it may be a useful reminder that access to air conditioning is not universal and that localized urban environments can be subject to extreme outdoor heat despite the weather station forecast.

Heat exposure patterns related to the dog's home environment were similarly apparent in the analysis. As expected, outdoor dogs had a greater overall risk for exposure to extreme heat. For urban outdoor dogs, heat exposure may be increased compared to their surroundings due to the increased number of reflective surfaces (i.e., buildings, roads, etc.) and decreased shade (26). Also as hypothesized, indoor dogs experienced the most consistent temperatures; however, as noted earlier, rural indoor dogs experienced slightly greater heat exposures at nighttime. As mentioned previously, this could be due to decreased access to air conditioning in our rural study population. Thus, outdoor rural dogs may benefit from increased air flow during nighttime hours to which indoor dogs may not have access.

From this analysis, we can make two general recommendations regarding the environmental risk for heat related morbidities for veterinarians to consider when advising dog owners on summer safety for their dogs. The first recommendation is that veterinarians should consider each owner's home as an individual environment. Consider whether the owner lives in a rural or urban environment, whether the dog is kept indoors or outdoors, and what environmental features could exacerbate or alleviate the dog's potential heat exposure. Some of this increased exposure may be in indoor rural dogs, who experience reduced air flow, particularly at night. Urban dogs, on the other hand, can be subjected to extreme heat exposure, potentially from reduced air flow outdoors and increased blacktop and reflective surfaces to concentrate solar radiation. These considerations lead to the second recommendation, which is to consider that the heat index was developed to represent the potential impact of heat exposure on humans, may not be representative of the local environment, and may underestimate the risks for our canine companions. Increased consideration to environmental risk factors may help alleviate individual risk factors for heat related morbidities in our pet dog populations.

This analysis provides an exploration of measuring environmental heat exposure in pet dogs and examining the potential impact of local environmental factors on their heat exposure. Nevertheless, there are several limitations to keep in mind when reviewing this analysis. First, our sample was small, with 30 dogs overall and 15 from each study site. This meant that any further stratification (i.e., rural vs. urban; indoor vs. outdoor; age; breed; etc.) produced small groupings. This reduced the ability to detect significant predictors in our regression analysis and made us less confident in the generalizability of findings, particularly for underrepresented populations (i.e., indoor rural dogs/outdoor urban dogs). Second, we had limited background information on study dogs as demographic data was self-reported and often incomplete or difficult to interpret (e.g., body condition and body weight, time outdoors, activity level, etc.). For example, brachycephalic dogs develop hyperthermia at ambient temperatures as low as 21°C, and body condition is known to worsen temperature regulation for these breeds (37, 38). Further, dichotomizing indoor/outdoor status based on reported hours outside may have oversimplified heat exposure patterns. For future studies, adding an in-person interview with the owner and dog present could help standardize the data collection.

Additionally, this study did not collect any clinical data. Because of this we cannot correlate the temperatures experienced with any physiological effects experienced by the dogs (i.e., heat stress or heat stroke). Further, as dogs were not supervised at all times, we do not know how their daily activities or interactions with their environment may have influenced their heat exposure. Also, there has been, to our knowledge, no known evaluation of the feasibility of using the Heat Index to understand a dog's experience of heat and humidity exposure and risk of heat-related illness.

Finally, our unadjusted data suggested that the body heat of the dogs influenced the temperature measurements recorded by the iButtons. The data from one supervised dog owned by one author of this paper was used to calculate adjustment factors to account for body heat and may not accurately reflect the impact of body heat from all dogs. Further these adjustment factors were applied to four periods of the day to account for different activities common in each period, but do not directly correspond with the activities of each individual study dog that may increase the influence of body heat (e.g., when a dog is sleeping and possibly curled up around the iButton vs. when a dog is standing or sitting and the iButton is hanging freely from the collar). Future studies would benefit from a more comprehensive survey of the influence of canine body temperature on iButton readings while trialing different placements on the dog and housings for the iButtons, including time spent in automobiles, and consideration of removal of the collar during nighttime to minimize body heat influence.

Future studies can build upon this work to better categorize the heat exposure patterns seen in the pet dog population. A benefit of our study design was that it was convenient for owners to participate and easy to implement in conjunction with the human study. However, with small adjustments more standardized data can be obtained. Instructing owners to complete diaries that roughly track their dog's activities throughout the day or utilizing pet cameras or monitors would provide additional detail on potential environmental factors that influence heat stress in dogs. Alternatively, a home environment assessment, examining access to shade, air conditioning, reflective or absorptive surfaces would provide a more individualized assessment of the dog's local environment.

## Conclusion

This study applies a novel method of measuring individually experienced temperatures in pet dogs and could be applicable to other veterinary species at risk of heat stress. As we have illustrated, small wearable thermometers can be used to measure individually experienced temperatures in pet dogs, enabling more specific assessment of environmental risks for heat related morbidities. Through these measurements we compiled a baseline of knowledge on the heat exposures of pet dogs and the environmental factors that augment or minimize those risks. Additionally, we demonstrated that traditional measures of heat exposure, such as the heat index from the nearest weather station, may underestimate the risks of excessive heat for dogs in both rural and urban, and indoor and outdoor environments. Nighttime heat index measurements were higher for dog measurements, indicating that opportunities for at risk dogs to obtain relief from the heat may be less prevalent than expected. The knowledge gained by these experiments can serve as a basis for future development of canine specific recommendations for minimizing the risk of heat stroke in pet dog populations. Future work should focus on expanding this knowledge through larger studies. As our global climate continues to warm, expanding this knowledge base may be useful in protecting our pets from the harmful effects of excessive heat.

## Data Availability Statement

The de-identified raw data supporting the conclusions of this article can be made available by the authors, provided all of the conditions of the Institutional Review Board and IACUC protocols for this research are maintained.

## Ethics Statement

The study involving human participants was reviewed and approved by the Virginia Tech IRB (Protocol #17-608). The human participants provided written informed consent to participate in this study. Pet dog participation was reviewed and approved by Virginia Tech IACUC (Protocol #17-134). Written informed consent was obtained from the pet owners for the participation of their dogs in this study.

## Author Contributions

KB conceived and designed the study with JG. KB and JG implemented the study and performed data collection. KM, SW, and JG analyzed and interpreted data. KM drafted the manuscript. All authors revised the manuscript for critically important intellectual content and approved the final version to be published.

## Funding

This work was supported by the National Institutes of Health [Grant number R01ES023029 (PI: JG)], the Virginia-Maryland College of Veterinary Medicine Summer Veterinary Student Research Program, and the Boehringer Ingelheim Veterinary Scholar program. These funding sources were not involved in study design, collection, analysis and interpretation of data, in the writing of the report, or in the decision to submit the article for publication.

## Conflict of Interest

The authors declare that the research was conducted in the absence of any commercial or financial relationships that could be construed as a potential conflict of interest.

## Publisher's Note

All claims expressed in this article are solely those of the authors and do not necessarily represent those of their affiliated organizations, or those of the publisher, the editors and the reviewers. Any product that may be evaluated in this article, or claim that may be made by its manufacturer, is not guaranteed or endorsed by the publisher.

## References

[1] Bell JE Langford Brown C Conlon K Herring S Kunkel KE Lawrimore J. Changes in extreme events and the potential impacts on human health. J Air Waste Manage Assoc. (2018) 68:265–87. 10.1080/10962247.2017.1401017 29186670 PMC9039910

[2] Rabaiotti D Woodroffe R. Coping with climate change: limited behavioral responses to hot weather in a tropical carnivore. Oecologia. (2019) 189:587–99. 10.1007/s00442-018-04329-1 30740614 PMC6418050

[3] Limaye VS Vargo J Harkey M Holloway T Patz JA. Climate change and heat-related excess mortality in the eastern USA. Ecohealth. (2018) 15:485–96. 10.1007/s10393-018-1363-0 30159651 PMC6572724

[4] Centers for Disease Control and Prevention. Extreme Heat; A Prevention Guide to Promote Your Personal Health and Safety [WWW Document]. Available online at: https://stacks.cdc.gov/view/cdc/7023 (2004).

[5] National Weather Service. Weather Related Fatalities and Injury Statistics, Year 2020. Available online at: https://www.weather.gov/hazstat/ (accessed June 11, 2021) (2021).

[6] Kuras ER Hondula DM Brown-Saracino J. Heterogeneity in individually experienced temperatures (IETs) within an urban neighborhood: insights from a new approach to measuring heat exposure. Int J Biometeorol. (2015) 59:1363–72. 10.1007/s00484-014-0946-x 25567543

[7] Mora C Dousset B Caldwell IR Powell FE Geronimo RC Bielecki CR. Global risk of deadly heat. Nat Clim Chang. (2017) 7:501–6. 10.1038/nclimate3322

[8] Vaidyanathan A Saha S Vicedo-Cabrera AM Gasparrini A Abdurehman N Jordan R. Assessment of extreme heat and hospitalizations to inform early warning systems. Proc Natl Acad Sci USA. (2019) 116:5420–7. 10.1073/pnas.1806393116 30833395 PMC6431221

[9] Shapiro Y Rosenthal T Sohar E. Experimental heatstroke: a model in dogs. Arch Intern Med. (1973) 131:688–92. 10.1001/archinte.1973.00320110072010 4701378

[10] Berman A Horovitz T Kaim M Gacitua H. A comparison of THI indices leads to a sensible heat-based heat stress index for shaded cattle that aligns temperature and humidity stress. Int J Biometeorol. (2016) 60:1453–62. 10.1007/s00484-016-1136-9 26817655

[11] Carter AJ Hall EJ. Investigating factors affecting the body temperature of dogs competing in cross country (canicross) races in the UK. J Therm Biol. (2018) 72:33–8. 10.1016/j.jtherbio.2017.12.006 29496012

[12] Gogolski SM O'Brien C Lagutchik MS. Retrospective analysis of patient and environmental factors in heat-induced injury events in 103 military working dogs. J Am Vet Med Assoc. (2020) 256:792–9. 10.2460/javma.256.7.792 32176578

[13] Bruchim Y Horowitz M Aroch I. Pathophysiology of heatstroke in dogs - revisited. Temperature. (2017) 4:356–70. 10.1080/23328940.2017.1367457 29435477 PMC5800390

[14] Stern A. Canine environmental hyperthermia: a case series. J Vet Med Sci. (2019) 81:190–2. 10.1292/jvms.18-0586 30555124 PMC6395217

[15] Bruchim Y Klement E Saragusty J Finkeilstein E Kass P Aroch I. Heat stroke in dogs: a retrospective study of 54 cases (1999-2004) and analysis of risk factors for death. J Vet Intern Med. (2006) 20:38–46. 10.1111/j.1939-1676.2006.tb02821.x 16496921

[16] Romanucci M Della Salda L. Pathophysiology and pathological findings of heatstroke in dogs. Vet Med Res Reports. (2013) 4:1. 10.2147/VMRR.S29978 32670838 PMC7337213

[17] Iampietro PF Fiorica V Higgins EA Mager M Goldman RF. Exposure to heat: comparison of responses of dog and man. Int J Biometeorol. (1966) 10:175–85. 10.1007/BF01426864 5978317

[18] Hanneman GD Sershon JL. A Temperature/Humidity Tolerance Index for Transporting Beagle Dogs in hot Weather. Civil Aeronautical Institute (1987). Available online at: https://rosap.ntl.bts.gov/view/dot/21294

[19] Potter AW Berglund LG O'Brien C. A canine thermal model for simulating temperature responses of military working dogs. J Therm Biol. (2020) 91:102651. 10.1016/j.jtherbio.2020.102651 32716889

[20] Dausmann KH. Measuring body temperature in the field—evaluation of external vs. implanted transmitters in a small mammal. J Therm Biol. (2005) 30:195–202. 10.1016/j.jtherbio.2004.11.003

[21] Bernhard MC Kent ST Sloan ME Evans MB McClure LA Gohlke JM. Measuring personal heat exposure in an urban and rural environment. Environ Res. (2015) 137:410–8. 10.1016/j.envres.2014.11.002 25617601 PMC4355189

[22] Wang S Wu CYH Richardson MB Zaitchik BF Gohlke JM. Characterization of heat index experienced by individuals residing in urban and rural settings. J Expo Sci Environ Epidemiol. (2021) 31:641–53. 10.1038/s41370-021-00303-x 33597724 PMC8273073

[23] Runkle JD Cui C Fuhrmann C Stevens S Del Pinal J Sugg MM. Evaluation of wearable sensors for physiologic monitoring of individually experienced temperatures in outdoor workers in southeastern U.S. Environ. Int. (2019) 129:229–38. 10.1016/j.envint.2019.05.026 31146157

[24] Heaviside C Macintyre H Vardoulakis S. The urban heat island: implications for health in a changing environment. Curr Environ Heal Rep. (2017) 4:296–305. 10.1007/s40572-017-0150-3 28695487

[25] Huang G Zhou W Cadenasso ML. Is everyone hot in the city? Spatial pattern of land surface temperatures, land cover and neighborhood socioeconomic characteristics in Baltimore, MD. J Environ Manage. (2011) 92:1753–9. 10.1016/j.jenvman.2011.02.006 21371807

[26] Tian Y Zhou W Qian Y Zheng Z Yan J. The effect of urban 2D and 3D morphology on air temperature in residential neighborhoods. Landsc Ecol. (2019) 34:1161–78. 10.1007/s10980-019-00834-7

[27] Wang S Richardson MB Wu CYH Zaitchik BF Gohlke JM. Effect of an additional 30 minutes spent outdoors during summer on daily steps and individually experienced heat index. Int J Environ Res Public Health. (2020) 17:7558. 10.3390/ijerph17207558 33080822 PMC7589302

[28] Richardson MB Chmielewski C Wu CYH Evans MB McClure LA Hosig KW. The effect of time spent outdoors during summer on daily blood glucose and steps in women with type 2 diabetes. J Behav Med. (2020) 43:783–90. 10.1007/s10865-019-00113-5 31677087

[29] Maxim Integrated. DS1922L: iButton Temperature Loggers With 8KB Data-Log Memory [WWW Document]. (2016). Available online at: https://www.maximintegrated.com/en/products/ibutton-one-wire/data-loggers/DS1922L.html (accessed December 21, 2020).

[30] Leys C Ley C Klein O Bernard P Licata L. Detecting outliers: do not use standard deviation around the mean, use absolute deviation around the median. J Exp Soc Psychol. (2013) 49:764–6. 10.1016/j.jesp.2013.03.013

[31] Grolemund G Wickham H. Dates and times made easy with lubridate. J Stat Softw. (2011) 40:1–25. 10.18637/jss.v040.i03

[32] Wickham H. ggplot2: Elegant Graphics for Data Analysis. New York, NY: Springer-Verlag (2016).

[33] Anderson GB Bell ML Peng RD. Methods to calculate the heat index as an exposure metric in environmental health research. Environ Health Perspect. (2013) 121:1111–9. 10.1289/ehp.1206273 23934704 PMC3801457

[34] R Core Team. R: A Language and Environment for Statistical Computing (2013).

[35] US Department of Commerce NOAA N.W.S. Heat Index [WWW Document]. Available online at: https://www.weather.gov/safety/heat-index (accessed December 21, 20).

[36] Hall EJ Carter AJ O'Neill DG. Incidence and risk factors for heat-related illness (heatstroke) in UK dogs under primary veterinary care in 2016. Sci Rep. (2020) 10:1–12. 10.1038/s41598-020-66015-8 32555323 PMC7303136

[37] Lilja-Maula L Lappalainen AK Hyytiäinen HK Kuusela E Kaimio M Schildt K. Comparison of submaximal exercise test results and severity of brachycephalic obstructive airway syndrome in English bulldogs. Vet J. (2017) 219:22–6. 10.1016/j.tvjl.2016.11.019 28093105

[38] Davis MS Cummings SL Payton ME. Effect of brachycephaly and body condition score on respiratory thermoregulation of healthy dogs. J Am Vet Med Assoc. (2017) 251:1160–5. 10.2460/javma.251.10.1160 29099251

