# Supplementary material for: Environmental Heat Exposure Among Pet Dogs in Rural and Urban Settings in the Southern United States
Source: Front Vet Sci. 2021 Oct 5;8:742926. doi: 10.3389/fvets.2021.742926 (PMC8525463; doi:10.3389/fvets.2021.742926)
Supplement: Supplementary file 1 [file Data_Sheet_1.pdf]

Supplemental File 1

Correction factor exploratory study methods, analysis and results

One dog (female, mixed breed, 15 yrs. old, 12.8 kg body weight) wore an iButton on her collar for 5 days with her indoor/outdoor times monitored. Two additional iButtons were placed inside the houses, at places where the dog spent most of her time. We calculated a mean of dog temperature and house temperature at each hour, and a temperature difference = dog's iButton temperature – house iButton temperature. We used this data to apply a correction factor for four daily periods of time. Our assumption for dogs in this study is that they were more active in early mornings and late afternoons, which led us to divide 24 hours into 4 am-10 am, 10 am-4 pm, 4 pm-10 pm, 10 pm-4 am by considering both dog activity patterns from participating dogs and Figure 1 results. The results are included in the manuscript as main results. Below we provide additional results using only 2 correction factors (daytime/nighttime).

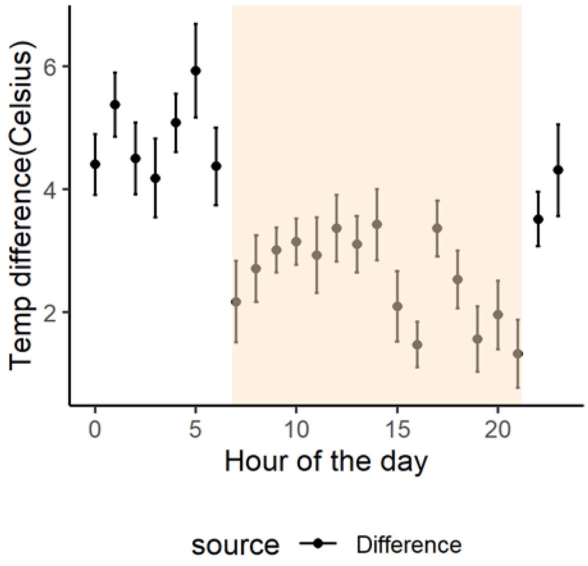

Data Sheet 1 Figure. Mean and 95%CI intervals of temperature differences of average hourly iButton on dog's collar temperature -iButtons in rooms of house.

Day/Night 2 correction factors

- In Figure 1, based on the temperature difference, we divided 24 hours into daytime = 7:00AM -9:59PM (yellow background) and night time = 10:00 PM -6:59AM.
- The mean temperature difference is 4.63 (95% confidence interval 4.42 to 4.83) with a standard error of 0.10 during nighttime (10:00 PM-6:59 AM) and 2.55 (95% confidence interval 2.41 to 2.69) with a standard error of 0.07 during the daytime (7:00AM - 9:59PM).

- We applied these 2 correction factors above in the data set. The results are added in the 2 (Day/night) column in Table 3 and Table 4 below. Overall, the results of using 2 correction factors and the results in the manuscript (4 correction factors) are similar, with the exception of the coefficient for WS in the heat index model (Table 4). The 2 correction factor model suggest significant and positive associations between the Heat Index reported from the nearest weather station and estimated Heat Index experienced by the dogs; whereas the 4 correction factor model does not suggest an association.

Table 3. Linear mixed effect model results with dog's individually experienced temperatures over 24 hours as the outcome.

Correction factors	4 (main results)	2 (Day/night)
Population	All	All
Fixed effects	β (95%CI)	β (95%CI)
Intercept	16.46 (14.93, 17.98)*	15.06(13.47,16.66)*
T[owner] (°C)	-0.023 (-0.059, 0.015)	-0.02(-0.06,0.02)
T[WS] (°C)	0.30 (0.23, 0.36) *	0.34(0.28,0.41)*
T[neighborhood] (°C)	0.14 (0.072, 0.21) *	0.14(0.07,0.21)*
Rural setting	-0.75 (-2.18, 0.68)	-0.78(-2.28,0.72)
Indoor	-1.21 (-2.64, 0.22)	-1.2(-2.7,0.3)
Rural setting: Indoor	0.25 (-2.08, 2.60)	0.24(-2.21,2.69)

'*' denotes a β estimate with a 95% confidence interval that does not contain 0. T = Temperature. WS= Weather Station.

Table 4. Linear mixed effect model results with dog's individually experienced HI in 24 hours as the outcome.

**Correction factors**	**4 (main results)**	**2 (Day/night)**
Population	All	All
Fixed effects	β (95%CI)	β (95%CI)
Intercept	28.32 (25.34, 31.32)*	25.95(22.87,29.03)*
HI[owner] (°C)	-0.052 (-0.093, -0.025) *	-0.05(-0.09,-0.01)*
HI[WS] (°C)	-0.045 (-0.057, 0.15)	0.11(0.01,0.21)*
HI[neighborhood] (°C)	0.11 (0.017, 0.19)*	0.11(0.02,0.2)*
Rural setting	-1.60 (-4.64, 1.43)	-1.68(-4.81,1.45)
Indoor	-1.71 (-4.75, 1.32)	-1.78(-4.91,1.35)
Rural setting: Indoor	1.28 (-3.67, 6.25)	1.39(-3.72,6.5)

‘*’ denotes a β estimate with a 95% confidence interval that did not contain 0. HI = Heat Index. WS = Weather Station.
