# Supplementary figures and images for: Environmental Heat Exposure Among Pet Dogs in Rural and Urban Settings in the Southern United States
Source: Front Vet Sci. 2021 Oct 5;8:742926. doi: 10.3389/fvets.2021.742926 (PMC8525463; doi:10.3389/fvets.2021.742926)

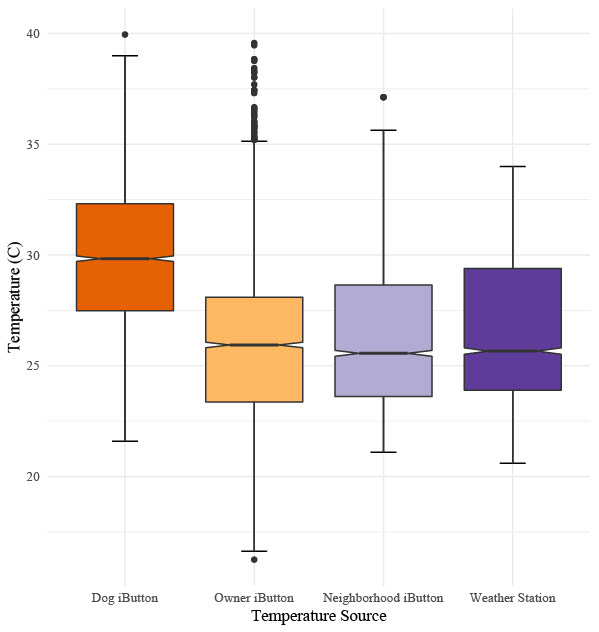

Supplement: Supplementary file 2 [file Image_1.JPEG]

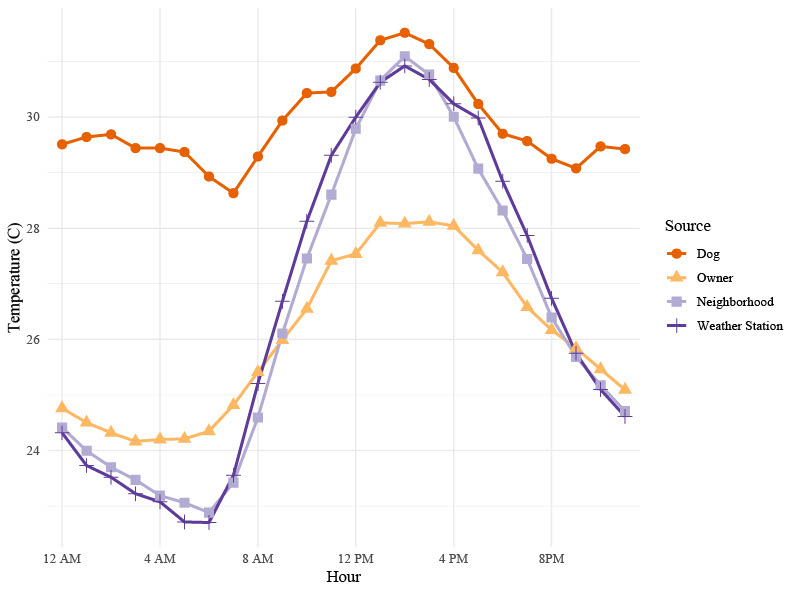

Supplement: Supplementary file 3 [file Image_2.JPEG]

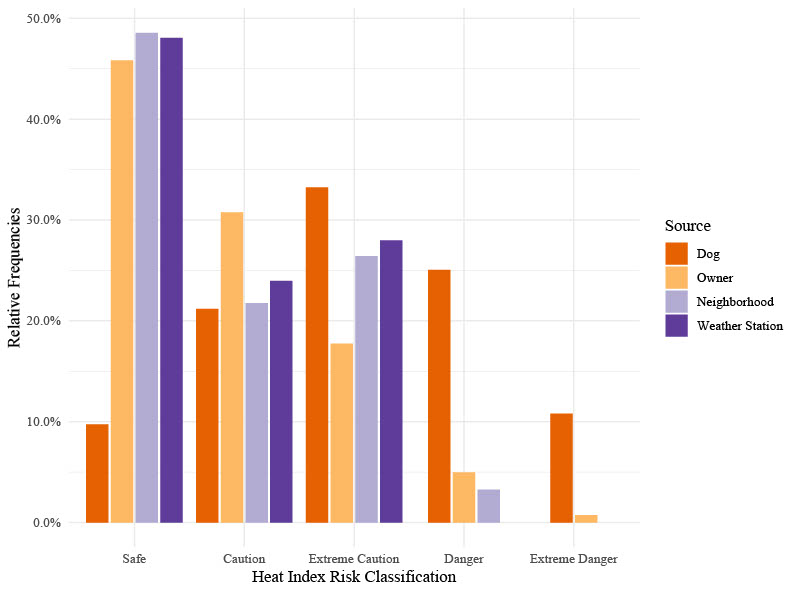

Supplement: Supplementary file 4 [file Image_3.JPEG]
